# Pepsin as a Marker of Reflux Aspiration in Children With Esophageal Atresia: A Pilot Study

**DOI:** 10.3389/fped.2020.00094

**Published:** 2020-03-19

**Authors:** Yadhavan Upendran, Steven T. Leach, Harveen Singh, James McBride, Paul S. Thomas, Yvonne Belessis, Usha Krishnan

**Affiliations:** ^1^School of Women's and Children's Health, University of New South Wales, Sydney, NSW, Australia; ^2^Department of Gastroenterology, Sydney Children's Hospital, Sydney, NSW, Australia; ^3^Department of Respiratory Medicine, Sydney Children's Hospital, Sydney, NSW, Australia; ^4^Department of Respiratory Medicine, Prince of Wales' Clinical School, Prince of Wales' Hospital, UNSW, Sydney, NSW, Australia

**Keywords:** pepsin, aspiration, oropharyngeal reflux, laryngopharyngeal reflux, esophageal atresia

## Abstract

**Background:** Reflux aspiration secondary to gastroesophageal reflux disease (GERD) is one of the causes of chronic gastrointestinal and respiratory morbidity in children with esophageal atresia (EA). Currently there are no simple, validated non-invasive tests for the diagnosis of reflux aspiration in children.

**Objectives:** The aim of this pilot study was to investigate pepsin detected in exhaled breath condensate (EBC) and saliva as a potential non-invasive marker of reflux aspiration in children with EA.

**Methods:** EBC and saliva samples were prospectively collected from children with EA aged between 5 and 18 years attending a multidisciplinary EA Clinic. Pepsin in the samples was assayed by two methods, a commercial lateral flow device, the Peptest™ and an enzyme-linked immunosorbent assay (ELISA) and correlated with validated gastrointestinal and respiratory symptom questionnaires and objective measures of GERD and respiratory function.

**Results:** EBC were collected from 18 children with EA, 15/18 also provided salivary samples. Pepsin was not detected in any of the EBC samples using the Peptest™ and only 1/14 (7.1%) samples by the ELISA. However, pepsin was detected in 33 and 83% of saliva samples when analyzed with Peptest™ and the ELISA respectively. Salivary pepsin levels were significantly higher in children with reflux symptoms or wheeze. Pepsin was detected by the Peptest™ in the saliva of 5/5 (100%) children with histological evidence of reflux esophagitis compared with 0/2 (0%) in children with normal histology (*p* = 0.048).

**Conclusions:** Salivary pepsin was detected in a large proportion of children with EA and was significantly associated with GERD symptoms or wheeze. The role of salivary pepsin as a potential non-invasive marker of reflux aspiration in children with EA needs further validation in future studies with larger cohorts.

## What is known

Children with esophageal atresia associated with trachea-esophageal fistula have known gastrointestinal and respiratory complications, including gastro-esophageal reflux disease and reflux aspiration.

Reflux aspiration is difficult to diagnose and currently there are no sensitive or specific tests for assessment.

Pepsin A is present in gastric secretions and therefore is a specific marker of reflux aspiration when detected in the airways.

## What is new

Salivary pepsin is a new method of detecting reflux aspiration in children with esophageal atresia associated with trachea-esophageal fistula.

Salivary pepsin significantly correlates with both gastro-esopheageal reflux symptoms and wheeze in children with esophageal atresia.

Histological evidence of reflux in EA patients significantly correlated with levels of salivary pepsin.

## Introduction

Esophageal atresia associated with tracheo-esophageal fistula (EA), is commonly associated with long-term gastrointestinal and respiratory morbidity including gastroesophageal reflux disease (GERD), esophageal strictures, recurrent respiratory tract infections, chronic cough and asthma (1). GERD resulting in reflux aspiration is one of the causes for respiratory complications in this cohort (2). GERD occurs in 22–45% of EA patients (3).

Timely detection and management of reflux aspiration is essential to prevent long-term respiratory morbidity in EA patients (2). However, the diagnosis of micro-aspiration of gastric fluid remains challenging. Whilst invasive tests such as endoscopy, pH monitoring and multi-channel impedance are effective diagnostic tools for reflux, there is no currently available sensitive, specific, validated test for assessing reflux aspiration (4).

Over the past decade, there has been growing interest in pepsin as a biomarker of reflux aspiration. Pepsin is secreted as the zymogen pepsinogen from gastric chief cells, which is then cleaved to produce the active pepsin A protein. Pepsin A is only present in gastric secretions and therefore is a specific marker of reflux aspiration when detected in the airways (5). An assay that detects pepsin A only has potential clinical utility as a marker of reflux aspiration (6). This has been shown in recent studies, which have detected pepsin in bronchoalveolar lavage (BAL) and tracheal aspirate (TA) samples of reflux patients (7–9). In a study by Krishnan et al. (10), pepsin detected in TA was suggested to be a reliable marker of reflux aspiration in children.

The detection of gastric pepsin in exhaled breath condensate (EBC) is a potential method of screening for reflux micro-aspiration. EBC involves condensation of water vapor from breath, which encompasses aerosolized particles of respiratory fluid, that may contain particles useful in the detection of the disease (11, 12). EBC is gaining popularity as a research tool due to being inexpensive, non-invasive and safe (11). However, its current role as a biomarker for reflux aspiration is still under evaluation. While in a study by Timms et al., pepsin detected in the EBC of adults with obstructive lung disease significantly correlated with both reflux symptoms and sputum pepsin (13), another study in adults with chronic obstructive pulmonary disease and bronchiectasis, found no correlation between EBC pepsin and multichannel intraluminal impedance and pH (MII-pH) results (14). To our knowledge, no study to date has assessed EBC pepsin as a biomarker of reflux aspiration in children including those with EA.

Detection of pepsin in saliva has previously shown promising results in the diagnosis of GERD and oropharyngeal and laryngopharyngeal reflux (LPR), with reported sensitivities and specificities comparable to endoscopy (15–17). However, there is limited evidence of the correlation between salivary pepsin and presence of pepsin in the airways. The aim of the current study, therefore, was to investigate gastric pepsin levels in EBC and saliva in children with EA using two different pepsin assays, and to assess their potential as a non-invasive markers of reflux aspiration in these children.

## Materials and Methods

This was a prospective pilot study of children with repaired EA, aged between 5 and 18 years attending the multi-disciplinary EA clinic at Sydney Children's Hospital. Children were recruited and assessed during the period, April 2016 and September 2016. Patients with a tracheostomy were excluded (18).

### Sample Collection and Storage

During a routinely scheduled clinic visit, EBC was collected as per American Thoracic Society/European Respiratory Society (ATS/ERS) recommendations using the refrigerated circuit, EcoScreen I (Jaeger Toennies, Germany) (18, 19). Saliva was collected from each participant directly into 15 mL sterile plastic tubes. In participants undergoing gastroscopy, the clinician also collected a TA sample. Patients had fasted for 6 h prior to the procedure.

### Pepsin Assays

Pepsin concentrations were initially quantified using Peptest™ (RD Biomed Ltd, UK), a colorimetric assay that contains two specific monoclonal antibodies against human pepsin A and has a lower limit of detection of 16 ng/mL. Samples below this threshold were considered to be unmeasurable and reported as 0 ng/mL. If pepsin was present in the sample, control and positive test lines would appear on the assay strip. A lateral flow device reader utilizing optical detection quantified the positive test line intensity. These intensity readings were then converted to pepsin concentrations (ng/mL) using standard curves (15).

EBC, saliva and TA samples with sufficient volume available following the Peptest™ assay, were also assayed using a sandwich ELISA kit (Wuhan Fine Biotech Co., Ltd, China). This kit is specific to Pepsin A (PGA3) with no significant cross-reactivity and a lower detection limit of 1.56 ng/mL. Hence is a more sensitive assay compared to Peptest™ for pepsin A detection.

Both Peptest™ and the ELISA are pepsin A specific but have different applications (20). Peptest™ is easy to perform in the clinician's room but is not as sensitive as the ELISA, which must be undertaken in a dedicated laboratory. The pepsin A levels in saliva, EBC and TA in children with reflux micro-aspiration are likely to be very low, therefore both tests were included in the study to assess agreement between the tests as well as clinical utility and validity of these pepsin assay methods. Samples were measured in duplicate by a single investigator blinded to the clinical status and lung function of the participants.

### Clinical Measures

Lung function tests performed included spirometry and lung clearance index (LCI) measured by multiple breath washout. Spirometry was performed according to ATS/ERS guidelines (21) and determined to be abnormal if the forced expiratory volume in 1 s (FEV_1_) and the forced vital capacity (FVC) were <80% of the predicted values (22). Elevated levels of LCI were considered to reflect increased ventilation inhomogeneity and peripheral airway disease (23).

MII-pH studies and endoscopic biopsies were performed to assess GERD. A trained histopathologist determined evidence of reflux esophagitis upon microscopic examination of each biopsy (24), while MII-pH data were analyzed both using automated software and manually by a single, trained gastroenterologist. Time from clinic visit to gastroscopy, pH-impedance, and lung function was presented as Median (Interquartile Range). Only results of gastroscopy, pH-impedance testing, and TA collection within 6 months of the clinic visit were included for analysis.

### Questionnaires

All study participants and their parents/guardians were asked to complete the gastrointestinal pediatric quality of life questionnaire (PedsQL). Scores were calculated as percentages, with 0% referring to a poor quality of life and 100% referring to a good quality of life (25). Parents were also asked to complete the Liverpool Respiratory Symptom Questionnaire (LRSQ), over the last 3 months. Higher scores are associated with an increased respiratory morbidity, with a maximum score of 128 (26). Patient reported symptoms for regurgitation, heartburn, vomiting, hematemesis/malena, chronic cough and, hoarse voice, recurrent chest infections, dental enamel erosion, chest pain, coughing/choking/gagging, dysphagia, increased hiccups or burping were ascertained through an institution specific GERD questionnaire administered to all patients/parents that attend EA clinic at Sydney Children's Hospital.

### Statistical Analysis

The data was analyzed using GraphPad Prism (GraphPad Prism version 7.0 for Windows, GraphPad Software, La Jolla CA USA). For all variables, the D'Agostino & Pearson omnibus normality test was utilized to measure any divergence from the Gaussian distribution. Due to the non-normal nature of the pepsin concentration data sets, non-parametric tests were utilized (Mann-Whitney *U*-test, Fisher's exact test, and Spearman's rank correlation coefficient). Data is presented as mean (SD) for variables with normal distribution and median (IQR) for variables with non-normal distribution. A *p*-value < 0.05 was considered significant.

## Results

### Patient Characteristics

In total, EBC was collected from 18 EA children aged between 6 and 16 years old, of which 15 also provided salivary samples. Fourteen participants and their parents completed the PedsQL and LRSQ questionnaires. However, one PedsQL was excluded from the study due to multiple unanswered questions. Median (IQR) time from clinic visit to endoscopy was 5 (0–7) days, likewise to MII-pH was 14 (7–21) days, and to lung function testing was 14 (7–30) days. The demographics of the study cohort are presented in Table 1.

**Table 1:** Patient demographics (*n* = 18).

**Sex**	
Male: *n* (%)	9 (50)
Female: *n* (%)	9 (50)
**Age (years)**	
Median (IQR)	8 (7–10)
Range	6–16
**Gestational age (weeks)**	
Mean (SD)	36.4 (3.2)
Range	28–41
Prematurity: *n* (%)	9 (50)
**EA-TEF Type:** ***n*** **(%)**	
A	4 (22)
C	14 (78)
≥1 Associated anomalies^a^: n (%)	10 (56)
**Weight z-score**	
Mean (SD)	−0.27 (0.95)
Range	−1.8 to 1.4
**Height z-score**	
Mean (SD)	−0.33 (0.96)
Range	−2.4 to 1.5

a *Associated anomalies of EA include vertebral anomalies, anal atresia, cardiovascular anomalies, renal anomalies and limb defects*.

A summary of the gastrointestinal (GI) and respiratory morbidity, details of current medications and prior surgical interventions of the study group can be found in Table 2.

**Table 2:** GI & respiratory morbidity (*n* = 18).

**GI symptoms (last month): *n* (%)**	
Regurgitation/vomiting	6 (33)
Food bolus impaction	5 (28)
**Respiratory symptoms (last month):** ***n*** **(%)**	
Hoarse voice	6 (33)
Chronic cough	10 (56)
Wheeze	8 (44)
Recurrent chest infections^a^	11 (61)
Respiratory admissions in last 12 months: *n* (%)	5 (28)
**Endoscopic findings (*****n*** **=** **9):** ***n*** **(%)**	
Reflux esophagitis	6 (67)
Eosinophilic esophagitis	4 (44)
MII-pH testing performed: *n* (%)	4 (22)
**Current medications:** ***n*** **(%)**	
Proton pump inhibitor (PPI)	15 (83)
Prokinetic	7 (39)
Azithromycin	4 (22)
**Additional surgery:** ***n*** **(%)**	
Fundoplication	9 (50)
Gastrostomy	8 (44)
**Strictures:** ***n*** **(%)**	
Strictures requiring dilatations ever	12 (67)
≥4 Strictures requiring dilatations ever	7 (39)
**Current feeding:** ***n*** **(%)**	
Oral	15 (83)
Gastrostomy	3 (17)

a *3 or more chest infections ever*.

### Pepsin Assays

#### EBC Pepsin

Pepsin was not detected in any of the 18 EBC samples using the Peptest™. Fourteen EBC samples were available for the ELISA with pepsin detected in only 1 (7%) sample.

#### Salivary Pepsin

**Figure 1:**
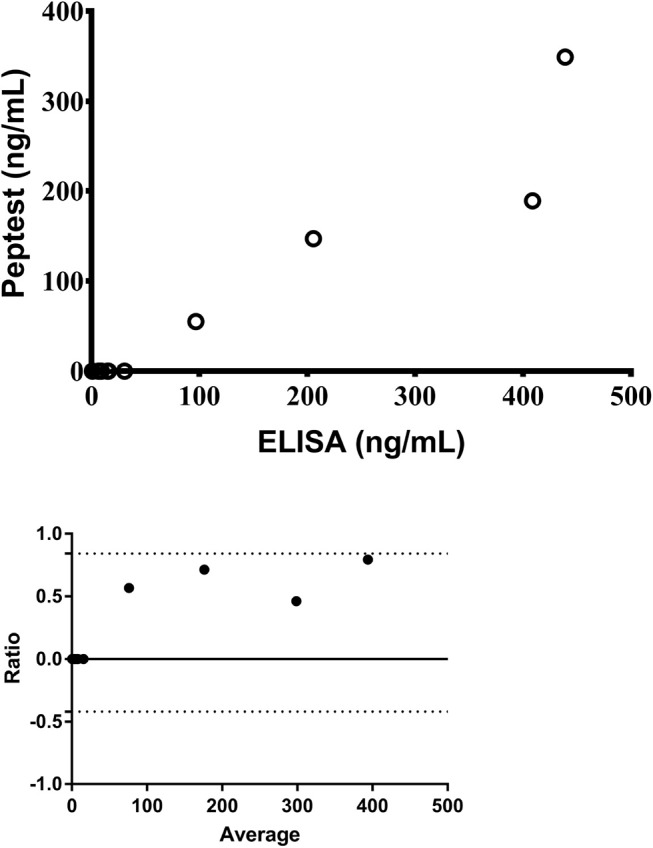
Correlation of salivary pepsin levels measured by Peptest™ and the ELISA **(top)** and Bland-Altman agreement between the two assays **(bottom)** (*n* = 12).

Pepsin was detected in 5/15 (33%) and 10/12 (83%) salivary samples, analyzed with the Peptest™ and the ELISA respectively. Saliva pepsin levels significantly correlated between the two methods [*r* = 0.84; 95% confidence interval (CI): 0.50–0.96; *p* < 0.0001] (Figure 1A) and the agreement between the Peptest™ and ELISA results is presented as a Bland-Altman plot [Bias = 0.21 (0.320; 95% limits of agreement from −0.42 to 0.84)] (Figure 1B). Even though there is good correlation between the two assays, the Bland-Altman plot indicates the ELISA returns a relatively higher result with higher concentrations.

#### Tracheal Aspirate Pepsin

**Figure 2:**
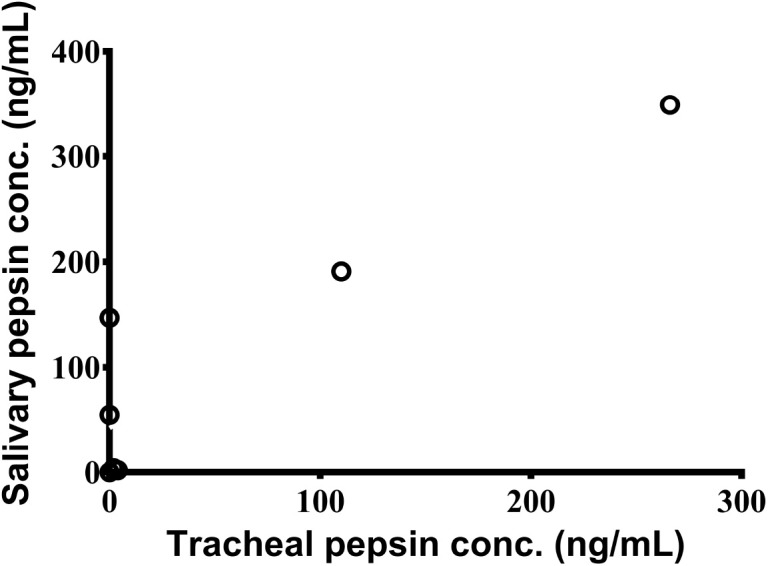
Significant association between pepsin concentrations in saliva and tracheal aspirates as measured by Peptest™ (*n* = 7).

TA samples were collected from 7 children. Pepsin was detected in 2/7 (29%) samples by the Peptest™ with TA pepsin levels significantly correlating with saliva pepsin levels (*r* = 0.83; 95% CI: 0.21–0.97; *p* = 0.024) (Figure 2). For the ELISA, pepsin was detected in all 3 (100%) TA samples.

Presence of pepsin in saliva did not significantly correlate with any of the MII-pH parameters that were assessed, including acid reflux index, number of reflux episodes, number of proximal episodes or symptom association with respiratory symptoms. There were also no correlations between salivary pepsin detection with spirometry or LCI results.

#### Objective Measures of GERD and Lung Function

Seven children underwent endoscopy with histological evidence of reflux esophagitis in 5 children and normal histology in 2 children. All endoscopy, except for 2 cases, were performed with patients on Proton Pump Inhibitors (PPI). Pepsin was detected by the Peptest™ in the saliva of 5/5 (100%) children with histological evidence of reflux esophagitis at a significantly higher rate than in children with normal histology, in whom pepsin was not detected 0/2 (0%) (*p* = 0.048). Similar results were obtained with the ELISA where salivary pepsin was higher in children with reflux esophagitis (307.3 ng/mL) compared to those with normal histology (12 ng/mL), although this was not a significant difference (*p* = 0.133).

#### Questionnaire Scores

**Figure 3:**
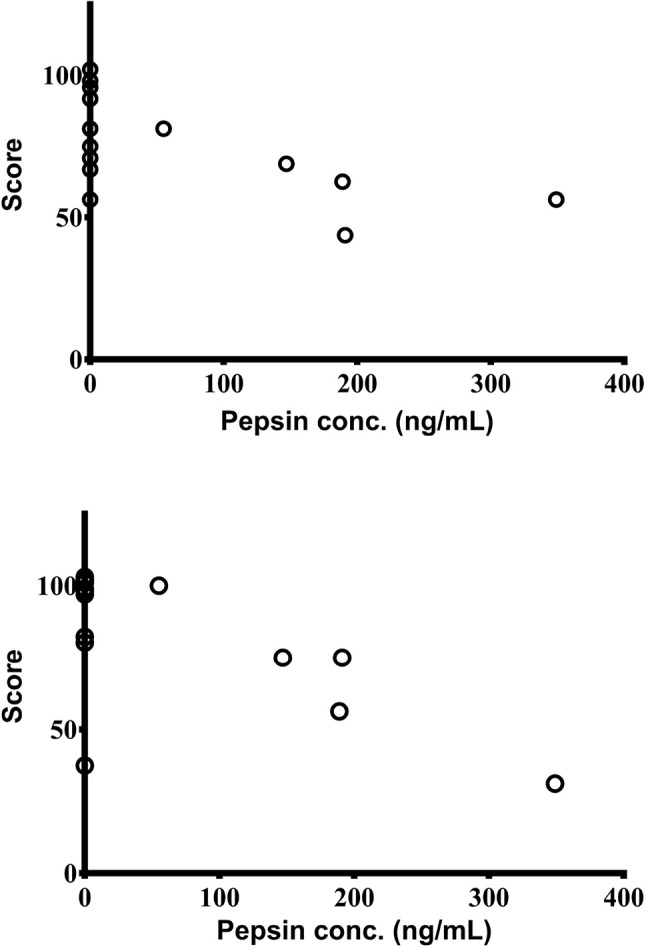
Significant association between salivary pepsin concentrations as measured by Peptest™ with scores from parent completed PedsQL sections; “Heartburn & Reflux” **(top)** and “Nausea & Vomiting” **(bottom)** (*n* = 14).

Overall PedsQL scores, and scores from the sub-sections “Heartburn & Reflux” (H/R) and “Nausea & Vomiting” (N/V), were compared with salivary pepsin concentrations. Pepsin levels (quantified by the Peptest™) negatively correlated with the sub-section scores from the parent completed PedsQL [H/R (*r* = −0.62; 95% CI: −0.87 to −0.12; *p* = 0.020) as well as N/V (*r* = −0.63; 95% CI: −0.87 to −0.13; *p* = 0.022)]. Higher pepsin concentrations were associated with lower scores and thus a poorer quality of life (Figures 3A,B). However, there were no significant relationships between saliva pepsin concentrations and overall parent PedsQL score or any of the child reported PedsQL and LRSQ scores.

#### Patient Reported Symptoms

**Figure 4:**
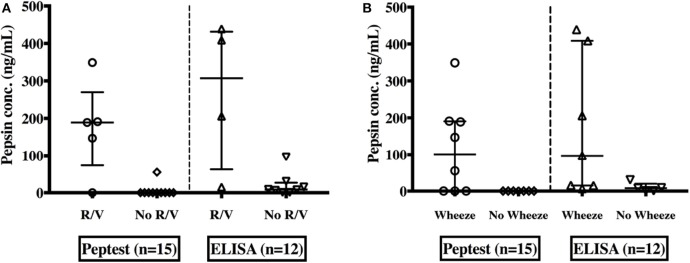
Pepsin concentrations in saliva as measured by both Peptest™ and the ELISA among EA children; with or without symptoms of regurgitation and/or vomiting (R/V) **(A)** and with or without a wheeze **(B)**.

Saliva pepsin concentrations were compared to reflux symptoms, GERD & lung function investigations. Children experiencing symptoms of regurgitation and/or vomiting had significantly higher pepsin detection rates (measured with Peptest™) [80 vs. 10%, (*p* = 0.017)] and significantly higher pepsin levels as measured by both assays, [189 ng/mL (73.5–270)] than their non-symptomatic peers [0 ng/mL (0–0)] (*p* = 0.0037), (measured by the Peptest™) and [307.3 ng/mL (62.6–431.5) vs. 8.57 ng/mL (2.22–26.9) (*p* = 0.026)], (measured with ELISA), as shown in Figure 4 compared to those without these symptoms.

In patients with wheeze, median pepsin concentrations were significantly higher [101 ng/mL (0–190.5) vs. 0 ng/mL (0–0)] (*p* = 0.026) by the Peptest™ and ELISA 96.8 ng/mL (14.9–408.8) vs. 8.07 ng/mL 0.8–19.9 (*p* = 0.047)] (Figure 4) compared to those with no wheeze. There was no significant difference in pepsin levels between children who experienced other respiratory symptoms (chronic cough, hoarse voice, recurrent chest infections) to those who did not.

## Discussion

Reflux aspiration of gastric contents secondary to severe GERD plays a significant role in the gastrointestinal and respiratory morbidity of children with EA. As reflux aspiration is only one of the causes of pulmonary aspiration, it is important to distinguish between the various causes of aspiration. However, it can be difficult to predict from history or observed feeding sessions whether patients have oropharyngeal dysphagia or GERD as the cause of their aspiration (27). Importantly, the developing lung of infants and young children with EA is vulnerable to lung injury and inflammation and it is paramount that both the lungs and airways are free from ongoing aspiration events. Recently, detection of pepsin in saliva and EBC has been proposed as a non-invasive test for laryngopharyngeal and pulmonary aspiration secondary to GERD. This pilot study aimed to investigate these simple non-invasive methods as sensitive markers of reflux aspiration in EA children.

### Salivary Pepsin

Salivary pepsin as a biomarker of GERD and LPR has been well-documented (5, 7–9). Salivary pepsin was detected in one-third (33%) of EA children in the current study using the Peptest™. This was lower rate than the study by Dy et al. (15), which reported a detection rate of 42% in children undergoing MII-pH testing for GERD. However, in the latter study only 48% of the patients were on acid suppression therapy compared to 83% on PPIs in the current study. Higher rates of detection of 50 and 68%, respectively have been reported in adults but both of these studies utilized post-prandial sampling when patients are more likely to be symptomatic, whereas the current study collected samples at the time of the clinic visit. The optimal timing of saliva collection is a subject of contention. Whilst some studies recommend multiple post-prandial sampling over 24-h (17), others suggest the need for sampling upon waking (28). Furthermore, adult patients spontaneously produce saliva. In contrast, younger patients can have difficulty in providing more than 0.5 mL of saliva, potentially lowering pepsin levels in the sample. Oral suctioning was utilized by Dy et al. (15) to produce larger sample volume for pediatric patients. However, this was not utilized in the current study due to the invasive nature and the potential for sample dilution with such a technique.

The current study reports that salivary pepsin concentrations were significantly higher in children with typical GERD symptoms of regurgitation and/or vomiting. Moreover, pepsin levels significantly correlated with parent reported PedsQL scores on reflux symptoms (heartburn, reflux, nausea, and vomiting). Similar to our study, a significant correlation between GERD symptoms and detection of salivary pepsin was also seen in a study by Farhath et al. where the rate of salivary pepsin presence in preterm infants with signs and symptoms of GERD was observed. Salivary pepsin (using mouth swabs) was detected in 72% infants with GERD compared to only 29% in infants without GERD (*P* < 0.001) (29).

In the current study, pepsin levels did not correlate with overall parent and child PedsQL scores. This is likely due to the presence of other questions in the survey about symptoms such as constipation, diarrhea, bloating, and stomach pain which are not relevant to a diagnosis of reflux.

In children with histological findings of reflux esophagitis, there were significantly more pepsin positive patients compared to those with normal histology. It is important to note, that all but 2 children were on PPI at time of endoscopy. This is in contrast to the study by Rosen et al., where there was no correlation between the presence of salivary pepsin and histological reflux esophagitis (30). However, the latter study was performed on normal children, whereas the current study looked at the EA cohort who often have a predisposition to severe GERD compared to normal children. Furthermore, whilst a tissue biopsy from endoscopy can assess for complications of GERD such as reflux esophagitis, it can not be used a measure of extra-esophageal complications of GERD such as reflux aspiration (30).

Similar to the study by Rosen et al., pepsin levels did not correlate with any of the parameters assessed by MII-pH testing (30). However, only 4 patients in the current study had undergone MII-pH studies. Previous studies by ourselves and others have also shown poor correlation between pepsin detected in tracheal and BAL with histological or MII-pH results (15, 31). Similar to our results and those by Rosen et al. and Dy et al. also found no association between MII-pH testing measurements and salivary pepsin, attributing this to the fact that MII-pH is a measure of esophageal reflux burden rather than the amount of reflux traveling into the oropharynx and airways (15). Other studies have shown different results with Fortunato et al. (32) finding significant correlations between salivary pepsin concentrations and multiple MII-pH parameters, especially proximal reflux events. Given these contradictory results, larger cohort studies are required to confirm correlation, if any, between MII-pH results and salivary pepsin levels.

In EA children with a wheeze, salivary pepsin was elevated compared to those without wheeze. However, salivary pepsin did not correlate with other respiratory symptoms, pulmonary function testing results or LRSQ scores. Although this could be due to the small sample size of the current study, a more likely explanation may be the multi-factorial etiology for respiratory morbidity in EA patients with reflux aspiration being only one of the causes (2).

### EBC Pepsin

The current study was unable to detect pepsin in any EBC samples obtained from children with EA using the Peptest™. Only one previous study conducted in adults with idiopathic pulmonary fibrosis, demonstrated positive EBC pepsin in 8.7% (2/23) of patients using the Peptest™ (33). Additional positive studies have detected pepsin in EBC, but the assays were not specific to human pepsin A. The inability of the current study to detect pepsin in EBC is likely to be due to several factors including: pediatric patients have smaller quantities of micro-aspiration, the lower sensitivity of both Peptest™ and the ELISA, and the assays utilized in the current study were specific to human pepsin whereas previous EBC studies used either porcine reagents or were not specific to pepsin A.

Currently, there are few reports of detectable pepsin in EBC samples in patients under the age of 18 years. A pediatric study of 47 patients with GERD, aged 2 to 14, found that pepsin was not detectable in any EBC samples (34). It is plausible that pediatric patients produce lower EBC volumes during similar sampling periods in comparison to adults. EBC pepsin as a marker of reflux aspiration relies on further development of more efficient and sensitive EBC collection methods.

### Limitations

In the current study, EBC pepsin was detected in only one patient and both the Peptest and ELISA assays lacked the sensitivity to detect pepsin in EBC. Therefore, there is a need for a specific assay with increased sensitivity for quantification of EBC pepsin in children.

Other limitations include the relatively small sample size and the fact that not all children had MII-pH testing in the current cohort, limited our ability to report associations of pepsin with impedance testing. However, MII-pH is only a measure of esophageal reflux burden rather than a marker for detection of gastric contents in the airways. The current study also lacked a healthy control group of normal children without GERD symptoms to establish control salivary and EBC pepsin levels. As other studies with a higher detection rate of salivary pepsin have collected targeted samples either at waking, after meals or at the time of symptoms, future studies should aim to collect salivary samples at similar times.

Regardless of these limitations, this study revealed promising results. Firstly, salivary pepsin was detected in significantly more EA children with GERD symptoms of regurgitation/vomiting and histological evidence of reflux esophagitis. There was also a significant correlation between concentrations of pepsin in saliva and TA collected from the same EA children. This indicates that higher pepsin levels in saliva may be predictive of the presence of tracheal pepsin in the airways secondary to reflux aspiration. This also suggests that salivary pepsin could potentially be used as a non-invasive marker of reflux aspiration in children with GERD. The higher prevalence of pepsin detection and levels of pepsin in children who had a wheeze further supports this association.

## Conclusions

Early and accurate diagnosis of reflux aspiration would have significant clinical implications in preventing chronic respiratory morbidity secondary to severe GERD in children with EA. The study shows that current specific methods of pepsin detection lack the sensitivity to detect pepsin in EBC and therefore more sensitive assays are needed. In contrast, has promise to detect both reflux and reflux aspiration in EA children and may have a role in clinical practice. As EA is a rare disease, larger multicentre collaborative studies are now required to validate the role of salivary pepsin as a surrogate marker of reflux aspiration.

## Data Availability Statement

The datasets generated for this study are available on request to the corresponding author.

## Ethics Statement

The studies involving human participants were reviewed and approved by Sydney Children's Ethics Committee. Written informed consent to participate in this study was provided by the participants' legal guardian/next of kin.

## Author Contributions

UK conceptualized the study, was involved in data collection and analysis, provided critical review of the original and subsequent manuscript drafts, and agreed to the final manuscript. YB, PT, JM, and SL were involved in data collection and analysis, provided critical review of the original and subsequent manuscript drafts, and agreed to the final manuscript. HS was involved in data collection, review of the original and subsequent revision of the manuscript. YU was involved in obtaining ethical approval, analyzing the data, and writing the original and subsequent revision of the manuscript.

### Conflict of Interest

The authors declare that the research was conducted in the absence of any commercial or financial relationships that could be construed as a potential conflict of interest.

## References

[1] Kovesi T Long-term respiratory complications of congenital esophageal atresia with or without tracheoesophageal fistula: an update. Dis Esophagus. (2013) 26:413–6. 10.1111/dote.12061 23679034

[2] Kovesi T. Aspiration risk and respiratory complications in patients with esophageal atresia. Front Pediatr. (2017) 5:62. 10.3389/fped.2017.00062 28421172 PMC5376561

[3] Krishnan U Mousa H Dall'Oglio L Homaira N Rosen R Faure C. ESPGHAN-NASPGHAN Guidelines for the evaluation and treatment of gastrointestinal and nutritional complications in children with esophageal atresia-tracheoesophageal fistula. J Pediatr Gastroenterol Nutr. (2016) 63:550–70. 10.1097/MPG.0000000000001401 27579697

[4] Little DC Rescorla FJ Grosfeld JL West KW Scherer LR Engum SA. Long-term analysis of children with esophageal atresia and tracheoesophageal fistula. J Pediatr Surg. (2003) 38:852–6. 10.1016/S0022-3468(03)00110-6 12778380

[5] Samuels TL Johnston N. Pepsin as a marker of extraesophageal reflux. Ann Otol Rhinol Laryngol. (2010) 119:203–8. 10.1177/000348941011900310 20392035

[6] Kahrilas PJ Kia L. Pepsin: a silent biomarker for reflux aspiration or an active player in extra-esophageal mucosal injury? Chest. (2015) 148:300–1. 10.1378/chest.15-0506 26238826

[7] Farrell S McMaster C Gibson D Shields MD McCallion WA. Pepsin in bronchoalveolar lavage fluid: a specific and sensitive method of diagnosing gastro-oesophageal reflux-related pulmonary aspiration. J Pediatr Surg. (2006) 41:289–93. 10.1016/j.jpedsurg.2005.11.002 16481237

[8] Starosta V Kitz R Hartl D Marcos V Reinhardt D Griese M. Bronchoalveolar pepsin, bile acids, oxidation, and inflammation in children with gastroesophageal reflux disease. Chest. (2007) 132:1557–64. 10.1378/chest.07-0316 17925430

[9] Ward C Forrest IA Brownlee IA Johnson GE Murphy DM Pearson JP. Pepsin like activity in bronchoalveolar lavage fluid is suggestive of gastric aspiration in lung allografts. Thorax. (2005) 60:872–4. 10.1136/thx.2004.036426 16055614 PMC1747219

[10] Krishnan U Mitchell JD Messina I Day AS Bohane TD. Assay of tracheal pepsin as a marker of reflux aspiration. J Pediatr Gastroenterol Nutr. (2002) 35:303–8. 10.1097/00005176-200209000-00012 12352517

[11] Hunt J. Exhaled breath condensate: an evolving tool for noninvasive evaluation of lung disease. J Allergy Clin Immunol. (2002) 110:28–34. 10.1067/mai.2002.124966 12110814

[12] Bajaj P Ishmael FT Exhaled breath condensates as a source for biomarkers for characterization of inflammatory lung diseases. J Anal Sci, Methods Instrum. (2013) 3:17–29. 10.4236/jasmi.2013.31004

[13] Timms C Thomas PS Yates DH. Detection of gastro-oesophageal reflux disease (GORD) in patients with obstructive lung disease using exhaled breath profiling. J Breath Res. (2012) 6:016003. 10.1088/1752-7155/6/1/016003 22233591

[14] Lee AL Button BM Denehy L Roberts S Bamford T Mu FT. Exhaled breath condensate pepsin: potential noninvasive test for gastroesophageal reflux in COPD and bronchiectasis. Respir Care. (2015) 60:244–50. 10.4187/respcare.03570 25352687

[15] Dy F Amirault J Mitchell PD Rosen R. Salivary pepsin lacks sensitivity as a diagnostic tool to evaluate extraesophageal reflux disease. J Pediatr. (2016) 177:53–8. 10.1016/j.jpeds.2016.06.033 27453366 PMC5037022

[16] Kim TH Lee KJ Yeo M Kim DK Cho SW. Pepsin detection in the sputum/saliva for the diagnosis of gastroesophageal reflux disease in patients with clinically suspected atypical gastroesophageal reflux disease symptoms. Digestion. (2008) 77:201–6. 10.1159/000143795 18617742

[17] Hayat JO Gabieta-Somnez S Yazaki E Kang JY Woodcock A Dettmar P. Pepsin in saliva for the diagnosis of gastro-oesophageal reflux disease. Gut. (2015) 64:373–80. 10.1136/gutjnl-2014-307049 24812000

[18] Horvath I Hunt J Barnes PJ Alving K Antczak A Baraldi E. Exhaled breath condensate: methodological recommendations and unresolved questions. Eur Respir J. (2005) 26:523–48. 10.1183/09031936.05.00029705 16135737

[19] Rosias PP Dompeling E Hendriks HJ Heijnens JW Donckerwolcke RA Jobsis Q. Exhaled breath condensate in children: pearls and pitfalls. Pediatr Allergy Immunol. (2004) 15:4–19. 10.1046/j.0905-6157.2003.00091.x 14998377

[20] Konstantinidi EM Lappas AS Tzortzi AS Behrakis PK. Exhaled breath condensate: technical and diagnostic aspects. Sci World J. (2015) 2015:435160. 10.1155/2015/435160 26106641 PMC4461795

[21] Miller MR Hankinson J Brusasco V Burgos F Casaburi R Coates A. Standardisation of spirometry. Eur Respir J. (2005) 26:319–38. 10.1183/09031936.05.00034805 16055882

[22] Pellegrino R Viegi G Brusasco V Crapo RO Burgos F Casaburi R. Interpretative strategies for lung function tests. Eur Respir J. (2005) 26:948–68. 10.1183/09031936.05.00035205 16264058

[23] Horsley A. Lung clearance index in the assessment of airways disease. Respir Med. (2009) 103:793–9. 10.1016/j.rmed.2009.01.025 19246184

[24] Sami SS Ragunath K The Los Angeles classification of gastroesophageal reflux disease. Video J Encycloped GI Endosc. (2013) 1:103–4. 10.1016/S2212-0971(13)70046-3

[25] Varni JW Kay MT Limbers CA Franciosi JP Pohl JF. PedsQL gastrointestinal symptoms module item development: qualitative methods. J Pediatr Gastroenterol Nutr. (2012) 54:664–71. 10.1097/MPG.0b013e31823c9b88 22008958

[26] Powell CV McNamara P Solis A Shaw NJ. A parent completed questionnaire to describe the patterns of wheezing and other respiratory symptoms in infants and preschool children. Arch Dis Child. (2002) 87:376–9. 10.1136/adc.87.5.376 12390904 PMC1763091

[27] Rosen R Vandenplas Y Singendonk M Cabana M DiLorenzo C Gottrand F Pediatric gastroesophageal reflux clinical practice guidelines: joint recommendation of the North American Society for Pediatric Gastroenterology, Hepatology, and Nutrition and the European Society for Pediatric Gastroenterology, Hepatology, and Nutrition. J Ped Gastroenterol Nutr. (2018) 66:516–54. 10.1097/MPG.0000000000001889 PMC5958910 29470322

[28] Na SY Kwon OE Lee YC Eun YG. Optimal timing of saliva collection to detect pepsin in patients with laryngopharyngeal reflux. Laryngoscope. (2016) 126:2770–3. 10.1002/lary.26018 27075393

[29] Farhath S He Z Saslow J Soundar S Amendolia B Bhat V. Detection of pepsin in mouth swab: correlation with clinical gastroesophageal reflux in preterm infants. J Matern Fetal Neonatal Med. (2013). 26:819–24. 10.3109/14767058.2013.764408 23311720

[30] Rosen R Johnston N Hart K Khatwa U Nurko S. The presence of pepsin in the lung and its relationship to pathologic gastro-esophageal reflux. Neurogastroenterol Motil. (2012) 24:129–33, e84–5. 10.1111/j.1365-2982.2011.01826.x 22141343 PMC3307906

[31] Safe M Cho J Krishnan U. Combined multichannel intraluminal impedance and pH measurement in detecting gastroesophageal reflux disease in children. J Pediatr Gastroenterol Nutr. (2016) 63:e98–106. 10.1097/MPG.0000000000001396 27574881

[32] Fortunato JE D'Agostino RB Jr. Lively MO. Pepsin in saliva as a biomarker for oropharyngeal reflux compared with 24-hour esophageal impedance/pH monitoring in pediatric patients. Neurogastroenterol Motil. (2017) 29:e12936. 10.1111/nmo.12936 27604397

[33] Fahim A Dettmar PW Morice AH Hart SP. Gastroesophageal reflux and idiopathic pulmonary fibrosis: a prospective study. Medicina. (2011) 47:200–5. 10.3390/medicina47040028 21829051

[34] Soyer T Soyer OU Birben E Kisa U Kalayci O Cakmak M. Pepsin levels and oxidative stress markers in exhaled breath condensate of patients with gastroesophageal reflux disease. J Pediatr Surg. (2013) 48:2247–50. 10.1016/j.jpedsurg.2013.02.100 24210194

